# Disruption of Learned Timing in P/Q Calcium Channel Mutants

**DOI:** 10.1371/journal.pone.0003635

**Published:** 2008-11-04

**Authors:** Akira Katoh, Peter J. Chapman, Jennifer L. Raymond

**Affiliations:** Department of Neurobiology, Stanford University, Stanford, California, United States of America; Vrije Universiteit Amsterdam, Netherlands

## Abstract

To optimize motor performance, both the amplitude and temporal properties of movements should be modifiable by motor learning. Here we report that the modification of movement timing is highly dependent on signaling through P/Q-type voltage-dependent calcium channels. Two lines of mutant mice heterozygous for P/Q-type voltage-dependent calcium channels exhibited impaired plasticity of eye movement timing, but relatively intact plasticity of movement amplitude during motor learning in the vestibulo-ocular reflex. The results thus demonstrate a distinction between the molecular signaling pathways regulating the timing versus amplitude of movements.

## Introduction

Motor learning can modify both the temporal properties and the amplitude of movements. However, the signaling pathways supporting these modifications are not fully understood. Here we report that the regulation of these two aspects of movement can be dissociated at the molecular level. Signaling through P/Q-type voltage-dependent calcium channels, which are highly expressed in the cerebellum, contributes selectively to the regulation of the temporal properties of movements, with a very limited role in the regulation of movement amplitude.

**Figure 1:**
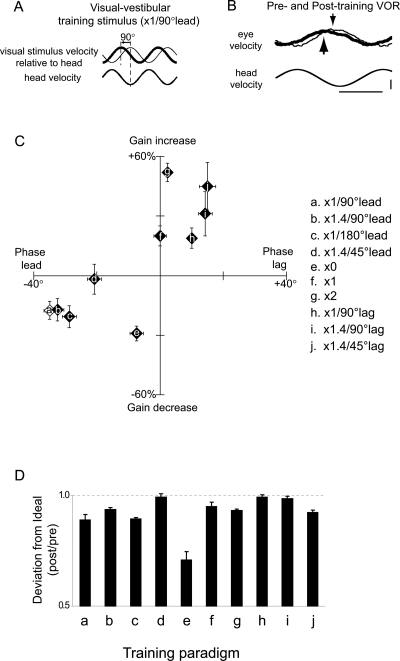
Changes in VOR gain and phase during motor learning in wild-type mice. A) One of the visual-vestibular training paradigms used to induce learning. The eye movement required to stabilize image motion is equal to the movement of the visual stimulus relative to the head. During x1/90°lead training, the visual stimulus movement had the same amplitude as head movement but was phase shifted to lead oppositely-directed head movement by 90° (thick upper trace). The eye movement required to stabilize an image under normal viewing conditions with an earth-stationary visual stimulus is also shown (thin upper trace). B) Representative traces illustrating the average VOR response to the same head velocity stimulus before (thin lines) and after (thick lines) 30 min of x1/90°lead training in a wild-type mouse. Arrows indicate the timing of peak eye velocity relative to peak head velocity before (downward arrows) and after (upward arrows) training. The training produced a shift in the time of peak eye velocity (VOR phase) and a decrease in the amplitude of the eye movement (VOR gain). Horizontal calibration bar indicates 500 ms; vertical bar indicates 10°/s for head velocity, 5°/s for eye velocity. C) Learned changes in the phase (abscissa) and gain (ordinate) of the VOR in wild-type mice induced by ten different visual-vestibular training paradigms (indicated by the letter on each symbol; see Methods for more detail). The training paradigm indicated by the open symbol, ‘a’, is used in Fig. 4. Error bars indicate standard error. D) Learned change in the deviation of the VOR response from the ideal eye movement required by each training paradigm to stabilize the visual image on the retina, calculated as (VOR_post_−Ideal)/(VOR_pre_−Ideal), where VOR_post_−Ideal and VOR_pre_−Ideal represent the length of the vector difference between the actual and ideal VOR gain and phase. A value less than 1 means that the change in the VOR during learning reduced image motion on the retina.

We used an oculomotor task in which the amplitude and temporal properties of movements are readily measured. Oculomotor reflexes, such as the vestibulo-ocular reflex (VOR), are typically tested using sinusoidal stimuli and characterized by the gain and phase of the eye movement response, which are measures of the peak amplitude and timing of the eye movement relative to the head movement, respectively. Both the gain and phase of the VOR can be adaptively modified through motor learning (Fig. 1).

Previous experiments have demonstrated that motor learning in the VOR is highly sensitive to the disruption of P/Q signaling. With severe disruption of P/Q signaling, there appear to be multiple, primary and secondary pathological processes, which can produce ataxia and severely disrupt many aspects of oculomotor performance, or execution, as well as motor learning [1], [2]. However, motor learning is most sensitive to disrupted P/Q signaling, as it is impaired in all P/Q mutant strains that have been tested, whereas only a subset of these strains exhibit additional deficits in baseline performance of the VOR, the optokinetic reflex (OKR) or the oculomotor integrator [1]–[3]. The P/Q mutants with the most restricted oculomotor deficit are hemizygous α1A subunit knockouts and heterozygous *leaner* mutants. α1A is the pore-forming subunit of the P/Q-type calcium channel, and *leaner* is a spontaneous point mutant of α1A. Both should express normal α1A subunits at approximately half the level of wild-type. Both mutants had no detectable motor phenotype on examination of complex motor behaviors, such as gait or performance on a rotorod [4], and both have normal baseline VOR and OKR [1]. However, the heterozygous *leaner* mutants and hemizygous α1A subunit knockouts exhibit a motor learning deficit—they undergo smaller changes in the VOR in response to reversed vision (“x(−1)”) training than wild-type mice [1] (see Materials and Methods for a detailed description of VOR motor learning paradigms). The learning deficit reported previously was a reduction in the changes in VOR phase induced by x(−1) training, however, since only a single training paradigm was used in that study, it was not clear whether regulation of the temporal properties of the eye movement was selectively impaired or whether there was a more general impairment of motor learning. Very few previous studies have analyzed the mechanisms supporting changes in the temporal properties of the VOR [5], [6], but have instead focused on changes in the gain of the VOR. Here, we used ten different VOR motor learning paradigms to directly compare the effect of disrupted P/Q signaling on the regulation of VOR phase versus gain by motor learning.

## Materials and Methods

### Animals

Experiments were performed on wild-type, α1A knockout hemizygous (α1A+/−) and *leaner* heterozygous (*tg^la^*/+) adult mice (10–20 wks old). α1A gene knockout mice were provided by Dr. Richard Tsien [7] and backcrossed to C57BL/6 for six generations. Hemizygous mutants and wild-type littermates were then obtained by intercrossing the hemizygotes. The C57BL/6-congenic *leaner* mutant strain with an oligosyndactylism marker gene (*Os +/+ Cacna1a^tg−la^*) was obtained from Jackson Laboratory (USA). *Leaner* heterozygous mice without the *Os* mutation were obtained by crossing *Os +/+ Cacna1a^tg−la^* mice with C57BL/6 mice, and C57BL/6 mice were used as controls. We found no significant difference between the C57BL/6 mice and the wild-type littermates from the hemizygous α1A crosses on eye tracking performance or motor learning in the VOR [1], and the results were also similar across control groups in the present study. Therefore results from the two control groups were pooled.

All animal protocols were approved by the Stanford University Administrative Panel for Laboratory Animal Care.

### Surgery

Surgical methods were identical to those described previously [8]. In summary, while the mouse was under anesthesia, a head post was attached to the top of the skull using anchor screws and dental acrylic, and a scleral search coil (IET, Marly, Switzerland) weighing ∼50 mg was implanted on the temporal side of the right eye beneath the conjunctiva. The search coil leads were run subcutaneously to a two-pin connector. Mice were allowed to recover from surgery for 5–7 days before oculomotor testing.

### General behavioral procedures

For experiments, the head of the mouse was immobilized by attaching the implanted head post to a restrainer. The restrainer was attached to a turntable (Carco IGTS, Pittsburgh, PA), which delivered a vestibular stimulus by rotating the mouse about an earth-vertical axis. Visual motion stimuli were delivered by a moving optokinetic drum made of a white translucent plastic half-dome with black and white vertical stripes, each of which subtended 7.5° of visual angle. The optokinetic drum was back-lit by two fiber optic lights (JH Technologies, San Jose, CA). The eye coil method [9], [10] was used to measure eye movements, as described previously [8]. The eye coil method was used because it is particularly reliable for measuring learning-related changes in the vestibulo-ocular reflex (VOR), since it allows stable and repeatable precision in the measurement of mouse eye movements, over time scales from milliseconds to days [8], [11]. Moreover, it allows measurement of the VOR in the absence of any illumination that could elicit visually driven eye movements [12]. Data were collected and stored as described previously [8].

After recovery from surgery, oculomotor performance was tested on two consecutive days using a range of vestibular and optokinetic stimuli. A day or more after the tests of oculomotor performance, motor learning was evaluated. Individual mice were run on multiple training paradigms. The number of animals we used for each training paradigm is presented in Table 1. To allow the VOR gain to return to baseline between experiments, mice were placed in their home cages in a normal visual-vestibular environment for at least 48 hours after an increase in VOR gain, and at least 72 hours after a decrease in VOR gain. These time periods were sufficient to allow the VOR gain to return to baseline [13]. The order of the training paradigms was pseudorandomized in each mouse.

**Table 1:** VOR motor learning paradigms.

Training Paradigm	a	b	c	d	e	f	g	h	i	j
Ideal gain	x1	x1.4	x1	x1.4	x0	x1	x2	x1	x1.4	x1.4
Ideal phase	90°lead	90°lead	180°lead	45°lead		0°	0°	90°lag	90°lag	45°lag
**No. of animals**										
wild-type	15	17	12	9	17	11	22	17	11	7
*tg^la^*/+	10	13	10	8	12	6	12	11	10	6
α1A+/−	9	12	9	8	11	8	9	9	11	7

Letters a–j for each training paradigm correspond to labels in Figs. 1–3 and Fig. S2. The notation describing each training paradigm denotes the eye movement gain and phase (relative to head motion) that would stabilize the image of the moving visual stimulus on the retina. x1/180°lead training is often referred to as x(−1) training.

### Training paradigms

We used ten different visual-vestibular training paradigms to evaluate motor learning (Table 1), designed to induce a range of different changes in VOR gain, VOR phase, or both. In all ten training paradigms, the vestibular stimulus was the same, with a sinusoidal angular head velocity profile at 1 Hz, ±10°/s. This vestibular stimulus was paired with 1 Hz sinusoidal motion of a visual stimulus, whose amplitude and phase relative to the vestibular stimulus depended on the training paradigm. To investigate the effects of training frequency on the learning impairments in the P/Q mutants, we tested one training paradigm (x1/90°lead, see below) at two additional stimulus frequencies, 0.5 and 2 Hz, with the same peak velocity used for 1 Hz training (±10°/s).

The notation describing each training paradigm denotes the eye movement gain and phase (relative to head motion) that would stabilize the image of the moving visual stimulus on the retina. The ideal eye movement is the same as the movement of the visual stimulus relative to the head. Normal viewing conditions, in which the visual stimulus is stationary in the world, require an eye movement that is equal in amplitude to the head movement (gain of 1) and exactly in phase with oppositely directed head movement (defined as a phase of 0°), and is hence referred to as x1/0°, or simply x1. To take another example, the x1.4/90°lead paradigm would require a VOR with a gain of 1.4 (larger than normal) and a phase such that eye velocity leads oppositely-directed peak head velocity by 90°.

Animals were exposed to the training stimulus for three 10-min blocks (30 min total). At the beginning of the experiment, and after each block of training, the VOR was tested in darkness with a ±10°/s sinusoidal vestibular stimulus at the training frequency (see Assessment of Learning below). The optokinetic reflex (OKR) was tested in the light using a ±10°/s sinusoidal visual stimulus at the training frequency before and after training in a subset of wild-type mice.

### Assessment of learning

Before and after each 10-min block of training, the VOR was assessed by turning the head-fixed animal in the dark, and measuring the eye movement response to the purely vestibular stimulus in three 30-sec blocks. Before each 30-sec VOR measurement block, a bell was rung to maintain animal alertness, followed by an 8-sec exposure to the vestibular stimulus in the dark before VOR measurement commenced. The VOR gain was calculated as the ratio of the eye to head velocity amplitudes. The VOR phase was calculated as the difference between the peak eye velocity phase and the peak head velocity phase in the opposite direction. A perfectly compensatory VOR would thus have a phase of zero. Learned changes in VOR gain were measured as a percentage of the initial VOR gain measured immediately before training. Learned changes in VOR phase were measured as the difference between the phase after versus before training. We also measured the gain and phase of the eye movements made in the presence of the visual-vestibular training stimulus, during the first one minute of training and at the end of training.

With 30 min of training, substantial changes in the VOR were induced, although the fully adaptive gain and phase were not achieved, even in wild-type mice. Longer training sessions were not used because of the difficulties associated with restraining mice for extended periods of time.

### Data analysis

Multiple cycles of head and eye velocity were aligned on the zero crossings of head velocity, and then averaged. Any cycle containing a saccade or motion artifact was excluded. Fourier analysis was used to extract the amplitude and phase of the eye movement from the averaged traces. To evaluate statistical differences between groups, we used analysis of variance (ANOVA) with post-hoc Scheffe's tests or with Bonferroni-corrected t-test, performed using StatView (SAS Inst., Cary, NC).

**Figure 2 pone-0003635-g002:**
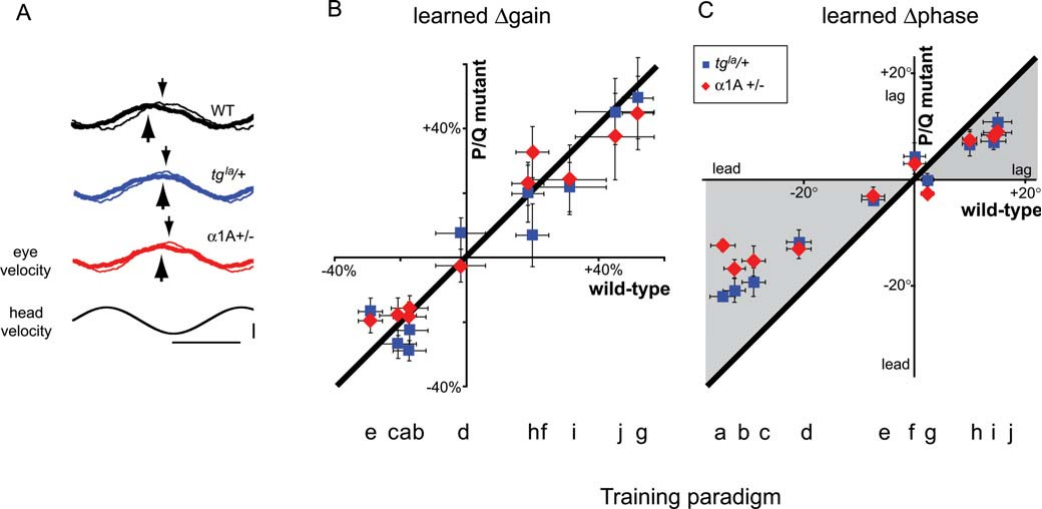
Changes in VOR gain and phase in P/Q mutant mice. A) Representative traces illustrating the VOR response to the same head velocity stimulus before (thin lines) and after (thick lines) 30 min of x1/90°lead training in a wild-type mouse (WT: black; same as Fig. 1B), a *leaner* heterozygous mouse (*tg^la^*/+: blue) and an α1A hemizygous mouse (α1A+/−: red). Horizontal calibration bar indicates 500 ms; vertical bar indicates 10°/s for head velocity, 5°/s for eye velocity. Average changes in B) VOR gain and C) VOR phase induced by each visual-vestibular training paradigm in the wild-type mice (abscissa) and the P/Q mutants (ordinate). The training paradigm is indicated by the letter below each set of corresponding data points (a–j, see Table 1). Error bars indicate standard error. See Table 1 for number of animals in each group.

For illustration purposes (Fig. 1B and 2A), representative raw eye velocity traces were obtained by averaging eye velocity within a sliding window of 100 ms. Sliding window averaging was not performed on any of the data used for statistical analyses.

## Results

To induce learning, we used ten different visual-vestibular training paradigms that were designed to produce a range of different learned changes in VOR gain and phase in wild-type mice (Fig. 1, see Materials and Methods for details). Motor learning in the VOR was tested by measuring the eye movement responses to head movements in total darkness, before and after training. In general, training altered the VOR so that the eye movements elicited by the vestibular stimulus in the dark were closer to the ideal eye movement response required to stabilize images in the presence of the visual-vestibular training stimulus (Fig. 1D). In a subset of wild-type mice, we also measured changes in the gain and phase of the optokinetic reflex (OKR), which shares some circuitry with the VOR. There was no correlation between the changes in OKR gain or phase and the changes in VOR gain or phase across the different training paradigms (Fig. S1), which suggests that the changes in VOR gain are not simply a reflection of plasticity in the OKR circuit.

Across the ten training paradigms, there was a correlation between the changes in VOR gain and the changes in VOR phase. Increases in VOR gain tended to be associated with decreases in phase lead (delayed timing of the peak eye movement response relative to the head movement) and decreases in the gain of the VOR tended to be associated with increases in phase lead (earlier peak eye movement) (Fig. 1C). This observation is consistent with models suggesting that learned changes in timing are linked mechanistically with changes in amplitude [14]–[16]. However, the learned changes in VOR phase and gain were not equally affected by the disruption of P/Q signaling (Fig. 2), indicating some independence of the signaling pathways supporting the regulation of these two aspects of the movement by motor learning.

Across the 10 training paradigms, learned changes in VOR gain were not sensitive to the disruption of P/Q signaling. Fig. 2B plots the changes in VOR gain induced by each training paradigm in *leaner* heterozygous mice (blue squares) and α1A hemizygous mice (red diamonds) as a function of the change induced by the same training paradigm in wild-type mice. The points lie near the 45° line of unity, indicating no significant difference in the modification of VOR gain by motor learning in the P/Q mutants as compared with wild-type (F_2, 299_ = 0.558, p>0.50, ANOVA).

In contrast to the normal learned changes in VOR gain, the changes in VOR phase induced by the ten training paradigms were significantly smaller in the *leaner* heterozygotes and α1A hemizygotes than in wild-type mice (Fig. 2C, gray quadrants; F_2, 299_ = 20.5, p<0.0001, ANOVA; p<0.0001 for α1A hemizygotes vs. wild-type, p<0.001 for *leaner* heterozygotes vs. wild-type, Scheffe's post-hoc test). Most of the training paradigms altered both the gain and phase of the VOR, hence the impaired changes in VOR phase were present even within the same training sessions that yielded normal changes in VOR gain (e.g., x1/90°lead training, Fig. 2, training paradigm ‘a’). This selective impairment of VOR phase adaptation was observed in both mice naïve to the training paradigms (Fig. S2) and in mice with previous experimental experience (Fig. 2). Thus, learned changes in VOR phase are more sensitive to the disruption of P/Q signaling than learned changes in VOR gain.

**Figure 3:**
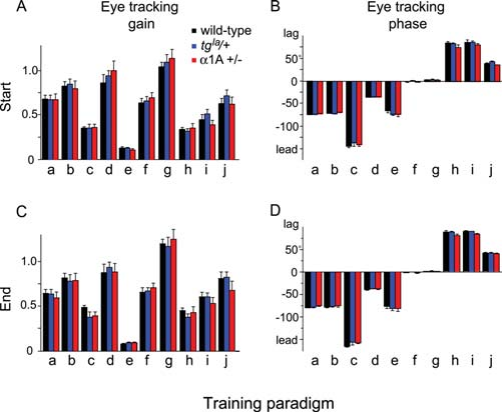
The gain (A, C) and phase (B, D) of the tracking eye movements relative to head movement made in the presence of each visual-vestibular training stimulus at the start (A, B) and end (C, D) of training. Letters correspond to the 1 Hz training paradigms described in Table 1. The tracking eye movements of the heterozygous *leaner* (blue) and hemizygous α1A mice (red) during training were not significantly different from wild-type mice (black).

The retinal image motion and other potential “error signals” experienced by the mice during training, such as visually-driven eye movements, were the same across genotypes. We examined the eye movements in the presence of the visual-vestibular stimuli used to induce learning (Fig. 3), and found no significant difference between the P/Q mutants and wild-type mice (pre-training: F_2, 299_ = 0.677, p>0.50 for eye movement gain, Fig. 3A; F_2, 299_ = 1.70, p>0.18 for eye movement phase, Fig 3B; post-training: F_2, 299_ = 0.569, p>0.50 for eye movement gain, Fig. 3C; F_2, 299_ = 0.518, p>0.50 for eye movement phase, Fig. 3D; ANOVA). This observation, combined with the normal baseline VOR and OKR performance of the *leaner* heterozygous and α1A hemizygous mice across a wide range of testing conditions (0.5–5 Hz, 5–25°/s) [1] indicates that their impairment results from perturbation of the neural processes involved in motor learning *per se*, rather than a secondary consequence of a sensory or motor performance deficit.

**Figure 4:**
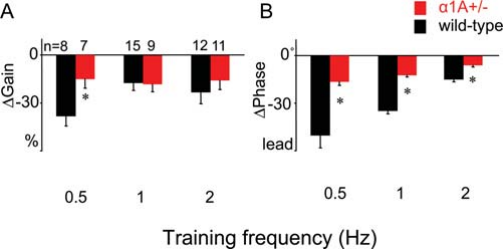
Effect of training frequency on the learned changes in VOR gain (A) and phase (B). Frequency-selective impairment of changes in VOR gain (A) and frequency-independent impairment of changes in VOR phase (B) in α1A+/− mice (red) relative to wild-type mice (black) during x1/90°lead training. Data for 1 Hz are the same as in Fig. 2 (training paradigm ‘a’). A different set of wild-type and α1A+/− mice was tested at 0.5 and 2 Hz. *: p<0.017 by Bonferroni-corrected t-test.

The data in Figs. 1–3 were obtained using visual and vestibular training and testing stimuli with a rotation frequency of 1 Hz. Several studies have suggested that different mechanisms are engaged when motor learning in the VOR is induced using different stimulus frequencies [17]–[21]. Therefore we tested the contribution of P/Q signaling to learning using higher and lower stimulus frequencies to determine whether the selective impairment of changes in VOR phase was present across stimulus frequencies in mice with disrupted P/Q signaling. These experiments employed the x1/90°lead training paradigm, which at 1 Hz induced a decrease in VOR gain and the most robust change in VOR phase of all training paradigms tested. In wild-type mice, x1/90°lead training at 0.5 or 2 Hz also induced a phase lead and decrease in VOR gain (Fig. 4, black bars). The α1A hemizygotes exhibited impaired phase adaptation at all frequencies tested (Fig. 4B, red bars; F_1, 56_ = 81.1, p<0.0001, ANOVA; p<0.005 at 0.5 Hz, p<0.0001 at 1 Hz and 2 Hz, Bonferroni-corrected t-test). Thus, disruption of P/Q signaling produces a general deficit in adaptive timing that can be observed across a range of training and testing conditions. At 1 and 2 Hz, there was no additional deficit in VOR gain adaptation, however at a lower frequency (0.5 Hz), gain adaptation was also significantly impaired (Fig. 4A; F_1, 56_ = 4.34, p<0.05, ANOVA; p<0.017 at 0.5 Hz, p>0.90 at 1 Hz, p>0.42 at 2 Hz, Bonferroni-corrected t-test). At all training frequencies, there was no significant difference between P/Q mutants and wild-type mice in the eye movements (Fig. S3A) or in the retinal image motion (Fig. S3B) in the presence of the visual-vestibular stimuli used to induce learning. Therefore, all deficits appear to be deficits in learning *per se*, rather than a sensory or motor performance deficit. The contribution of P/Q signaling to learned changes in VOR gain induced with low- but not high-frequency training supports the hypothesis that different neural mechanisms are recruited at different training frequencies. More generally, our results demonstrate that P/Q signaling contributes selectively, but not exclusively, to learned changes in the temporal properties of the VOR.

## Discussion

For many years, motor learning in the VOR was thought of as a unitary process. However, recent studies have revealed multiple, mechanistically distinct components of motor learning in the VOR. In particular, there is evidence for different plasticity mechanisms supporting increases versus decreases in VOR gain [13], [17], [22], [23] and different mechanisms supporting learning induced with low- versus high-frequency training stimuli [13], [20], [21]. For example, gain increases are selectively impaired in mice deficient in CaMKIV or αCaMKII, with sparing of gain decreases [17], [22]. Moreover, in the CaMKIV knockout mice, this deficit was shown to be present only for high-frequency training, and not low-frequency training [17]. Thus, different, molecularly-distinct mechanisms appear to support different components of motor learning, and these are differentially recruited by different visual-vestibular training paradigms.

The present results extend this new model of cerebellum-dependent learning by providing the first evidence for a molecular distinction between the signaling pathways supporting the modification of the temporal properties versus the amplitude of movements by motor learning. There is a component of motor learning in the VOR, which is highly sensitive to disruption of P/Q signaling, and which contributes selectively, although not exclusively, to changes in the temporal properties of the VOR.

Whenever a neural circuit is manipulated, one should consider the possibility that compensatory changes have occurred. In this study, the P/Q mutants may have developed compensatory mechanisms to overcome some of the effects of abnormal P/Q signaling. Moreover, it is conceivable that such compensatory mechanisms could contribute differentially to the recovery of VOR gain versus phase adaptation. Nevertheless, the inability of any compensatory process to rescue VOR phase adaptation in the P/Q mutants indicates that learned changes in VOR phase are more dependent on P/Q signaling than changes in VOR gain.

**Figure 5:**
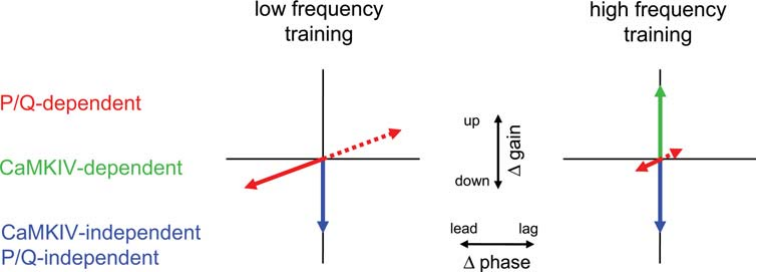
Working model of the multiple, molecularly-distinct components of motor learning in the VOR. The ordinate indicates changes in VOR gain and the abscissa indicates changes in VOR phase. A P/Q-dependent component of learning supports gain decreases linked with phase leads or gain increases linked with phase lags (red arrows). A CaMKIV-dependent component supports gain increases (green arrow). A CaMKIV- and P/Q-independent component supports gain decreases (blue arrows). The CaMKIV-dependent component (green arrows) is recruited more effectively by high-frequency training stimuli, whereas the P/Q-dependent component (red arrows) is recruited more effectively by low-frequency training stimuli.

Fig. 5 presents a working model of the components of cerebellum-dependent learning, and their recruitment during high- and low-frequency training, based on current and previous results. There are at least three components of learning: a CaMKIV-dependent component (green arrows in Fig. 5), which supports gain increases (possibly LTD of the parallel fiber–Purkinje cell synapses); a P/Q-dependent component (red arrows), which supports gain decreases linked with phase leads or gain increases linked with phase lags; and a CaMKIV- and P/Q-independent component (blue arrows), which supports gain decreases. The CaMKIV-dependent component (green arrows) is recruited more effectively by high-frequency training stimuli since the CaMKIV knockout mice showed the gain increase deficits only for high-frequency training [17], whereas the P/Q-dependent component (red arrows) is recruited more effectively by low-frequency training.

At all training frequencies, the phase changes depend on the P/Q-dependent component, hence the P/Q mutants exhibit consistently impaired phase changes. During low-frequency training, the P/Q-dependent component also makes a significant contribution to the gain changes, so both phase and gain changes are impaired in P/Q mutants undergoing low-frequency training. In contrast, during high-frequency training, the P/Q-dependent component makes a smaller contribution to learning, and accounts for a small fraction of the gain changes, rendering gain changes relatively insensitive to disruption of P/Q signaling.

The components of the model in Fig. 5, based on the results from genetically-modified mice, map remarkably well onto the components of a previous model, based on monkey physiology, which suggested that learning in the VOR involved both coordinated changes in the weights of the signaling pathways in the VOR circuit, which contribute selectively to changes in VOR gain, and changes in a time constant in the VOR circuit, which contribute to learned changes in the VOR gain and temporal properties [14]. The mouse data extend this previous model by mapping the components of learning onto specific molecular signaling mechanisms and by revealing that these components are differentially recruited at different training frequencies.

The frequency range at which gain changes transition from being P/Q-dependent to P/Q-independent is between 0.5 Hz and 1 Hz. Notably, it is in the very same frequency range that gain changes transition from being CaMKIV-independent to CaMKIV-dependent [17]. Electrophysiological analysis in monkeys suggests that the recruitment of different plasticity mechanisms by high- versus low-frequency training reflects the neural “instructive signals” available. The climbing fiber input to the cerebellum carries instructive signals appropriate to guide learning during both low- and high-frequency training, whereas the Purkinje cell simple spikes carry useful instructive signals only during low-frequency training [21]. Since P/Q-dependent mechanisms contribute to learning primarily during low-frequency training, this component of learning may be induced by instructive signals carried by the Purkinje cells, whereas the P/Q-independent components may be induced by instructive signals carried by the climbing fibers. This raises the possibility that the P/Q-dependent component involves plasticity in the vestibular nuclei/deep cerebellar nuclei, where instructive signals in the Purkinje cells have been hypothesized to act [24].

At the behavioral level, the timing of the instructive signals available to guide learning also varies with training stimulus frequency. Retinal image motion and/or the tracking eye movements present during training, and their relationship to the vestibular stimulus, are thought to provide the instructive signals guiding learning. The amplitude of retinal image motion and eye velocity, as well as their phase relative to the head movement, was similar during x1/90°lead training at 0.5 Hz as compared with 1 Hz (Fig. S3). However, the *timing* of peak eye movement or retinal image movement relative to head movement differed by hundreds milliseconds at the two frequencies. For example, the phase of the retinal slip in wild-type mice was 124.1±6.3° lead relative to peak head velocity at 1 Hz and 127.8±13.7° lead at 0.5 Hz, which represent 345 ms at 1 Hz and 710 ms at 0.5 Hz. This extra time shift of several hundred milliseconds could cause some plasticity mechanisms to drop out at frequencies below 1 Hz, making learning more reliant on a P/Q-dependent mechanism.

The inhibition of P/Q-type calcium channels can affect a number of neuronal functions, depending on the extent to which P/Q signaling is disrupted [25]. One neuronal function that is particularly sensitive to altered P/Q signaling is the precise timing of spikes. Purkinje cells from *leaner* heterozygotes in slice [26] and in *tottering* homozygotes *in vivo* [3] exhibit less regular spike timing than Purkinje cells in wild-type mice. The parallel between the high sensitivity of both spike timing and learned changes in the temporal properties of movements to disrupted P/Q signaling raises the intriguing possibility that spike timing dependent plasticity regulates the temporal properties of the VOR. Notably, the induction of LTD at the parallel fiber–Purkinje cell synapses, which has been the focus of most studies of cerebellum-dependent learning, should not be sensitive to the few milliseconds of jitter in spike timing observed in P/Q mutants, since LTD at these synapses can be effectively induced within a much broader timing window of hundreds of milliseconds between parallel fiber and climbing fiber activity (climbing fiber activation leading parallel fiber activation by tens of milliseconds or lagging by a few hundred milliseconds) [27]–[29]. Therefore it will be interesting to determine whether other synapses in the cerebellum and related circuitry have plasticity mechanisms that are sensitive to the few milliseconds of spike time jitter caused by reductions in P/Q signaling.

Finally, it is possible that P/Q signaling does not contribute directly to the plasticity mechanisms supporting VOR motor learning, but to the generation of a neural representation of timing upon which plasticity can act. Computational models have suggested that networks of granule cells and Golgi cells create a “menu” of differently-filtered versions of the input signal to the cerebellar cortex [15], [16]. The adaptive modification of movement timing would then involve the selection, through synaptic plasticity, of the filtered version of the signal with the appropriate temporal properties. If P/Q signaling contributed to the generation of differently filtered versions of the vestibular signals driving the VOR, representing the range of potential VOR phases, then disruption of P/Q signaling could selectively disrupt the ability of motor learning to adjust the phase, but not the gain of the VOR, as observed.

## Supporting Information

Figure S1 The relationship between the learned changes in A) VOR gain and OKR gain, B) VOR gain and OKR phase, C) VOR phase and OKR gain and D) VOR phase and OKR phase in wild-type mice, induced by the 10 visual-vestibular training paradigms. There was no significant correlation in any pair of changes in the VOR and OKR (R^2^ = 0.56, 0.01, 0.37 and 0.20 in A-D, respectively). Error bars indicate standard error.
(0.30 MB PDF)

Figure S2 Average changes in A) VOR gain and B) VOR phase induced by each visual-vestibular training paradigm in experimentally naïve animals; wild-type mice on the abscissa and the P/Q mutants on the ordinate, as in Fig. 2. The training paradigm is indicated by the letter below each set of corresponding data points (a–j, see Table 1). Error bars indicate standard error. No data are available for x1.4/90°lead training in *tgla*/+.
(0.33 MB PDF)

Figure S3 A) The gain (left) and phase (right) of the tracking eye movements relative to head movement during x1/90°lead training at different training frequencies. Across training frequencies, there was no significant difference between α1A hemizygotes and wild-type mice at the start (top; F_1, 56_ = 0.134, p>0.70 for gain, F_1, 56_ = 1.76, p>0.19 for phase, ANOVA) or end (bottom; F_1, 56_ = 0.233, p>0.60 for gain, F_1, 56_ = 2.09, p>0.13 for phase) of training. B) The peak velocity (left) and phase (right) of retinal image velocity (slip) at the start (top; F_1, 56_ = 0.011, p>0.90 for velocity, F_1, 56_ = 0.461, p>0.49 for phase) and end (bottom; F_1, 56_ = 1.29, p>0.26 for velocity, F_1, 56_ = 0.421, p>0.50 for phase) of training. Error bars indicate standard error.
(0.34 MB PDF)
