# Supplementary figures and images for: Disruption of Learned Timing in P/Q Calcium Channel Mutants
Source: PLoS One. 2008 Nov 4;3(11):e3635. doi: 10.1371/journal.pone.0003635 (PMC2572847; doi:10.1371/journal.pone.0003635)

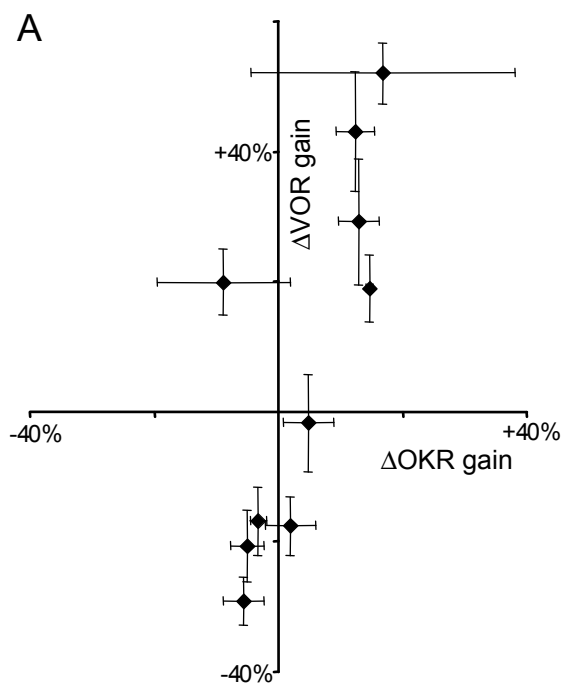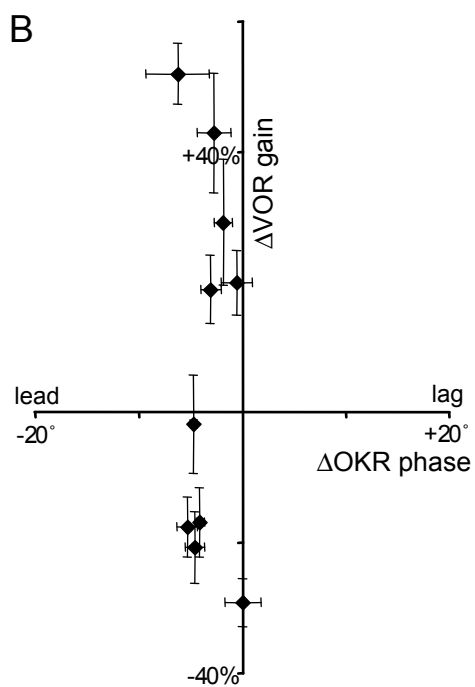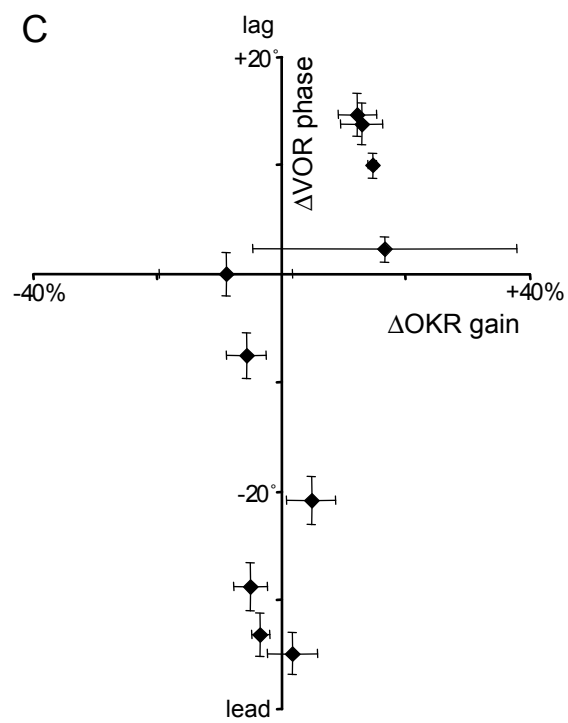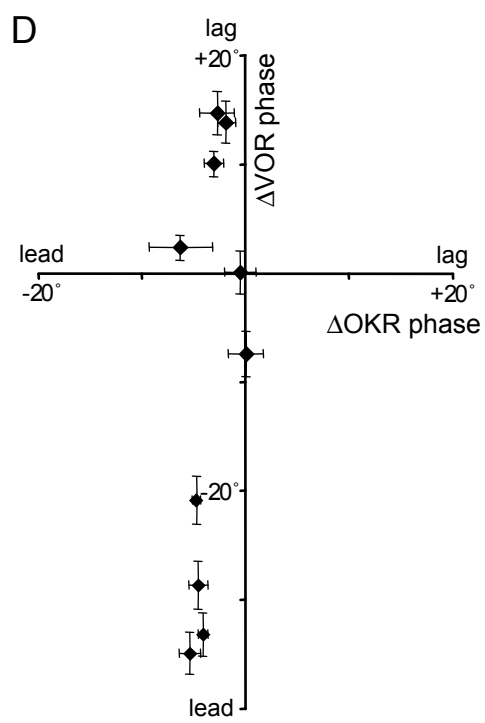

**FIGURE S1**

Supplement: Figure S1 — The relationship between the learned changes in A) VOR gain and OKR gain, B) VOR gain and OKR phase, C) VOR phase and OKR gain and D) VOR phase and OKR phase in wild-type mice, induced by the 10 visual-vestibular training paradigms. There was no significant correlation in any pair of changes in the VOR and OKR (R2 = 0.56, 0.01, 0.37 and 0.20 in A-D, respectively). Error bars indicate standard error. (0.30 MB PDF) [file pone.0003635.s001.pdf]

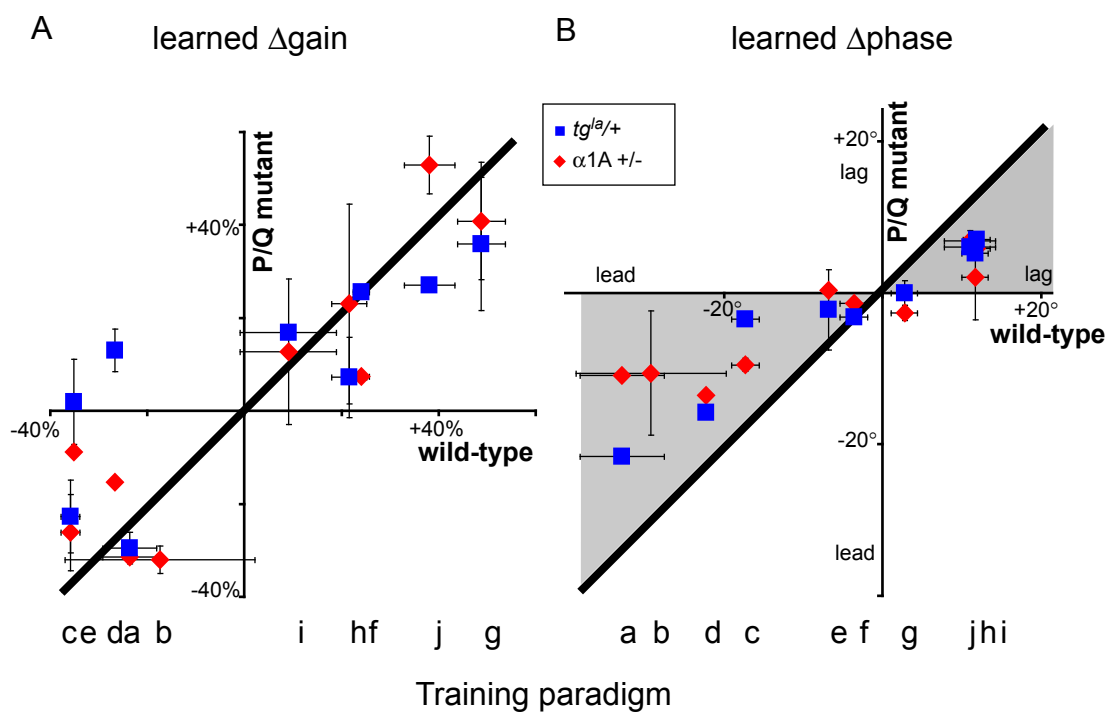

**FIGURE S2**

Supplement: Figure S2 — Average changes in A) VOR gain and B) VOR phase induced by each visual-vestibular training paradigm in experimentally naïve animals; wild-type mice on the abscissa and the P/Q mutants on the ordinate, as in Fig. 2. The training paradigm is indicated by the letter below each set of corresponding data points (a–j, see Table 1). Error bars indicate standard error. No data are available for x1.4/90°lead training in tgla/+. (0.33 MB PDF) [file pone.0003635.s002.pdf]
