# Supplementary material for: Disruption of Learned Timing in P/Q Calcium Channel Mutants
Source: PLoS One. 2008 Nov 4;3(11):e3635. doi: 10.1371/journal.pone.0003635 (PMC2572847; doi:10.1371/journal.pone.0003635)
Supplement: Figure S3 — A) The gain (left) and phase (right) of the tracking eye movements relative to head movement during x1/90°lead training at different training frequencies. Across training frequencies, there was no significant difference between α1A hemizygotes and wild-type mice at the start (top; F1, 56 = 0.134, p>0.70 for gain, F1, 56 = 1.76, p>0.19 for phase, ANOVA) or end (bottom; F1, 56 = 0.233, p>0.60 for gain, F1, 56 = 2.09, p>0.13 for phase) of training. B) The peak velocity (left) and phase (right) of retinal image velocity (slip) at the start (top; F1, 56 = 0.011, p>0.90 for velocity, F1, 56 = 0.461, p>0.49 for phase) and end (bottom; F1, 56 = 1.29, p>0.26 for velocity, F1, 56 = 0.421, p>0.50 for phase) of training. Error bars indicate standard error. (0.34 MB PDF) [file pone.0003635.s003.pdf]

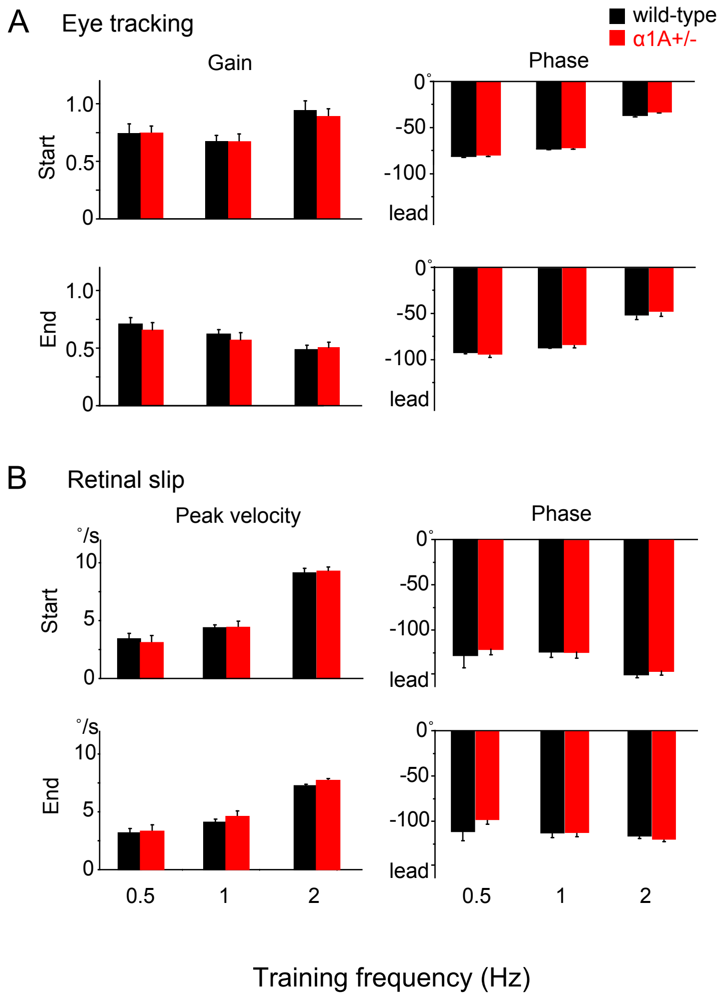

A
Eye tracking
wild-type
α1A+/-
Gain
Phase
Start
End
1.0
0.5
0
0°
-50
-100
lead
B
Retinal slip
Peak velocity
°/s
10
5
0.5
1
2
Training frequency (Hz)

FIGURE S3
